# Antenatal pentoxifylline therapy to prevent endotoxin-induced fetal injury in the preterm goat model

**DOI:** 10.4274/tjod.galenos.2020.19794

**Published:** 2020-12-10

**Authors:** Mekin Sezik, Afşin Köker, Özlem Özmen, Mehmet Halıgür, Duygu Kaşıkcı, Ahmet Aydoğan, Orhan Özatik

**Affiliations:** 1 Süleyman Demirel University Faculty of Medicine, Department of Obstetrics and Gynecology, Isparta, Turkey; 2 Burdur Mehmet Akif Ersoy University Faculty of Veterinary Medicine, Department of Obstetrics and Gynecology, Burdur, Turkey; 3 Burdur Mehmet Akif Ersoy University Faculty of Veterinary Medicine, Department of Pathology, Burdur, Turkey; 4 Çukurova University Faculty of Ceyhan Veterinary Medicine, Department of Pathology, Adana, Turkey; 5 Isparta University of Applied Sciences Faculty of Agriculture, Department of Animal Science, Isparta, Turkey; 6 Kütahya Health Sciences University Faculty of Medicine, Department of Histology and Embryology, Kütahya, Turkey

**Keywords:** Animal model, endotoxins, fetal brain injury, neuroprotection, pentoxifylline, preterm birth

## Abstract

**Objective:**

Pentoxifylline (PTX) has immunomodulatory properties and is known to reduce sepsis-associated infant mortality. We aimed to evaluate maternal oral and intra-amniotic administration of PTX for the prevention of fetal inflammation and injury in a caprine model.

**Materials and Methods:**

Inflammation-mediated fetal injury was induced with maternal granulocyte-colony stimulating factor and intra-amniotic endotoxin at 0.76 of gestation in date-mated pregnant goats. Eight groups were formed (n=4 each): Control, fetal injury, oral 30 mg/kg/day and 60 mg/kg/day PTX for 15 days + fetal injury, intra-amniotic 400 mg/kg and 800 mg/kg estimated fetal weight single-dose PTX with and without fetal injury. Preterm delivery by hysterotomy was performed at 0.80 of gestation to evaluate the fetal and placental effects. Immunochemistry for various markers including interleukins, caspases, cyclooxygenases, vimentin, myelin basic protein, and surfactant proteins were carried out in the fetal lungs, fetal brain, and placenta. Fetal plasma and amniotic fluid interleukins were also evaluated. Kruskal-Wallis H test and Mann-Whitney U test were used for comparisons.

**Results:**

High-dose (60 mg/kg/day) maternal prophylactic oral treatment attenuated endotoxin-related histological injury and was related to low inflammatory marker expressions comparable to the controls (p>0.05 except cyclooxygenase 2). Following maternal oral administration, fetal plasma and amniotic fluid levels of the studied interleukins were also lower than the untreated endotoxin-exposed animals (p<0.05 for all comparisons). Intra-amniotic PTX was associated with inconsistent results and increased inflammatory markers in some fetuses.

**Conclusion:**

Oral PTX before preterm birth mitigates intrauterine inflammation with neuroprotective effects in the fetus. PTX can be considered as a candidate drug for fetal brain injury prevention in the preterm period.

**PRECIS:** Using an inflammation-mediated fetal injury caprine model, we demonstrated neuroptotective effects of maternal pentoxifylline before preterm delivery.

## Introduction

Preterm birth is the most important cause of perinatal morbidity and mortality. There is growing evidence that subclinical intra-amniotic (IA) microbial colonization and inflammation is responsible for a significant number of preterm deliveries. Up to 40% of pregnancies that resulted in preterm deliveries were found to have positive inflammatory markers in the second-trimester amniotic fluid^(1)^. Moreover, increased subclinical IA inflammation is suggested to affect the fetus, with the inflammation probably commencing from the lungs and then propagating to the vulnerable fetal brain. This condition has been called as Fetal Inflammatory Response syndrome (FIRS) and is an indirect consequence of IA microbial colonization, most likely in an ascending manner through the cervix^(2,3)^. Ultimately, the outcome will be fetal brain injury, which may manifest itself as cerebral palsy (CP) in the long term^(4)^. Although peripartum hypoxia has conventionally been accused for the development of neonatal encephalopathy, data controlling for confounders show that most cases are indeed associated with antenatal insults to the developing fetal brain^(5)^.

Given the medical and social negative consequences of fetal brain injury, effective antenatal treatments are needed. Magnesium sulfate has been shown to have neuroprotective effects^(6)^, and many clinicians now routinely administer magnesium sulfate treatment before imminent early preterm delivery. However, some recent data have also indicated that magnesium sulfate may not be neuroprotective in the setting of chorioamnionitis^(7)^. Hence, targeted therapies that have a potential to specifically inhibit initial steps of the inflammatory cascades in the amniotic fluid and the chorioamniotic membranes may be required to decrease fetal brain injury. Moreover, use of fetal neuroprotective agents in a combined form in relatively low cumulative doses may demonstrate a cocktail effect, decreasing the overall side effects to the fetus, and with various therapeutics acting on different steps of inflammation.

Pentoxifylline (PTX) is a synthetic methylxanthine derivative that competitively inhibits phosphodiesterases with nonsteroidal immunomodulatory activities. Phosphodiesterase inhibition is believed to be associated with an anti-inflammatory effect, leading to decreased proinflammatory cytokine activity including tumor necrosis factor-alpha (TNF-α) and interferons (IFN)^(8)^. On this basis, PTX is presently under clinical investigation against neonatal sepsis, and a Cochrane meta-analysis has shown decreased sepsis-associated neonatal mortality with use of PTX as an adjunct to antibiotics^(9)^. A recent meta-analysis has also confirmed reduced mortality (relative risk, 0.50 and 95% confidence interval, 0.29 to 0.88) in sepsis following neonatal PTX treatment^(10)^. Therefore, maternal use of PTX for inflammation-driven pregnancy complications that might have effects on the fetus seems plausible, considering likely negligible toxicity to the preterm fetus.

In the sheep model, PTX was reported to decrease serum pro-inflammatory cytokines following experimentally induced endotoxemia^(11)^. Another experimental study revealed that PTX was partly effective against pre-eclamptic-like symptoms in ewes^(12)^. Considering these preliminary animal data, PTX can be considered to have fetal neuroprotective effects by mitigating IA inflammation. Moreover, IA injection of PTX can be hypothesized to deliver the drug in a target-specific manner to act on the fetal alveolar capillary bed and the gastrointestinal system by fetal swallowing.

The experimental ovine and caprine pregnancy models have been in use for translational research due to the physiological similarities with the human pregnancy, avoidance of multiple pregnancies with proper mating strategies, feasibility of IA administrations and sampling, and availability of adequate amount of fetal blood and tissues. Hence, preterm goat model may offer some advantages for experimental obstetric research.

The aim of the current study was to evaluate maternal oral and IA administration of PTX for the prevention of fetal inflammation and injury in a caprine model. We hypothesized that both oral and IA routes of PTX therapy were effective against inflammation-mediated fetal injury in the preterm period, particularly in the developing lung and brain tissues. To test this hypothesis, we used the small ruminant experimental model that utilized maternal granulocyte-colony stimulating factor (G-CSF) followed by high-dose IA endotoxin to aggravate intrauterine inflammation. We then evaluated various inflammatory, apoptotic, and injury parameters in the amniotic fluid, fetal blood plasma, placenta, fetal lungs, and fetal brain of the preterm goat fetuses.

## Materials and Methods

### Animal Material

Süleyman Demirel University Animal Experimentation Local Ethics Committee approved the study protocol (approval number: 15/03, date: 23.08.2011). Particular national regulations and principles of laboratory animal care were followed. A priori power analysis was conducted to test the difference between two independent group means for a 50% decrease or increase in the mean, using a two-tailed test and an alpha of 0.05. Result showed that 4 animals in a group were required to achieve a power of 80%. Therefore, the experimental preterm goat model included 32 date-mated singleton pregnant hair goats (Capra hircus) with 8 groups (n=4 each) formed at day 100 of gestation (term pregnancy, approximately 150 days). The animals were reared on pasture and/or standard food, given water and mineral salts ad libitum, and were sheltered in a semi-open pen. All experiments were carried out at the Faculty of Veterinary Medicine, Mehmet Akif Ersoy University. Maternal age and prepregnancy body weight was 4-5 years and 40±5 kg, respectively.

### Experimental Groups

The experiments were carried out in 2 phases. The first phase included validation of inflammation-mediated preterm fetal injury experimental model, including the vehicle control (group 1, n=4) and the positive control (group 2, n=4) groups. Details and results of these first 2 groups with proper validation of the novel modified experimental model initially defined by Watanabe et al.^(13)^ have been described previously^(14)^. Recombinant G-CSF at a daily dose of 50 microg (dissolved in 2 mL normal saline) (Neupogen Roche, F. Hoffmann-La Roche Ltd, Basel, Switzerland) to induce low-grade maternal inflammation was injected into maternal jugular vein for 5 consecutive days (gestational days, 110-114) with controls receiving intrajugular normal saline. At gestational day 115, amniocentesis with a 20-gauge amniocentesis needle (EchoTip, Cook Medical, United States) was performed under ultrasound guidance (Echo Camera SSD-500, Aloka, Tokyo, Japan) with exclusion of inappropriate allantoic entry by the color and viscosity of the fluid^(15)^. Amniotic fluid, watery in consistency and pale amber in color was aspirated from the inner amniotic sac close to the fetus. Using the amniocentesis needle in the amniotic sac, high-dose (20 mg) IA endotoxin (lipopolysaccharides from Escherichia coli O55:B5, L 2880, Sigma-Aldrich, Missouri, USA) was used to induce IA and fetal inflammation in group 1, while the positive controls (group 2) received identical amount of IA normal saline through amniocentesis. Validation of the model with resultant necrotizing funisitis associated with abundant leukocyte infiltration leading to necrotic arc formation in the vascular wall of the umbilical vessel and secondary fetal brain injury was shown in all of the fetuses (n=4) in the experimental group (group 2, positive controls)^(14)^. Priming with maternal G-CSF followed by high-dose (20 mg) endotoxin closely mimics the FIRS, in which low-grade preclinical systemic maternal and IA inflammation is followed by a relatively abrupt insult that leads to fetal brain injury that is evident following endotoxin exposure^(14)^.

The second phase of the experiments included treatment (PTX) groups to test the oral as well as IA efficacy and safety of antenatal PTX therapy. Group 3 and group 4 were designed to assess the prophylactic use of oral PTX at 2 different doses against subsequent inflammatory-driven fetal injury. Does in both of these groups (n=4 each) were started on oral PTX at day 100, and daily treatment continued for 15 days from gestation day 100 to 114 at a daily dose of 30 mg/kg maternal weight (low dose) and 60 mg/kg maternal weight (high dose) PTX, respectively. A mortar and pestle were used to grind commercially available tablets (Trental CR 600 mg film tablet, Sanofi Aventis, Istanbul, Turkey) into a uniform powder. After weighing on precision scales to calculate the dose, the PTX powder was dissolved in 10 mL of sterile water and administered to the does via an oral feeding catheter connected to a 10 mL-syringe. The does were observed for 10 minutes postadministration, and the procedure was repeated if the solution was not properly swallowed or spitted out by the animal. Animals in other treatment groups (groups 5-8) were given 10 mL of sterile water without PTX. We determined the daily low- and high-doses of oral PTX, depending on previous pharmacokinetic data from large animals^(16,17)^. Group 3 and 4 animals also received G-CSF (days 100-114) and IA endotoxin to induce fetal injury similar to positive controls.

Group 5 and group 6 aimed to examine the therapeutic effectiveness of single-use IA PTX at 2 different doses against concurrent inflammation-mediated fetal injury. Pregnant does in group 5 (n=4) and group 6 (n=4) received the 5-day G-CSF regimen and high-dose IA endotoxin as previously defined. Additionally at day 115, either single low-dose (400 mg/kg estimated fetal weight) or high-dose (800 mg/kg estimated fetal weight) PTX was injected into the amniotic fluid following endotoxin administration, using an amniocentesis needle under ultrasound guidance. Estimated fetal weight was typically considered as 1.5 kg, corresponding to 600 mg (30 mL) and 1.200 mg (60 mL) of IA single-dose administration of PTX (Trentilin ampoule 100 mg/5 mL, Santa Farma, Istanbul, Turkey). Dose calculation for IA administration depended on the assumption that around 2.5% of IA administered inert substances would be swallowed by the fetus within 1 hour^(18)^, corresponding to 10 mg/kg fetal weight and 20 mg/kg fetal weight for the low- and high-dose regimens, respectively in line with the recommended oral pediatric doses for PTX^(19)^.

The last 2 groups (group 7 and group 8) included use of IA PTX without intrauterine inflammation, i.e. non-administration of G-CSF and endotoxin. These groups assessed the safety and fetoplacental effects of IA administration of standalone PTX in the absence of intrauterine inflammation. Does in group 7 and group 8 received 2 mL of normal saline into the maternal jugular vein for 5 days followed by amniocentesis at gestational day 120 with PTX injections of 400 mg/kg estimated fetal weight (group 7) or 800 mg/kg estimated fetal weight (group 8) PTX.

### Induction of Preterm Delivery

At gestational day 120 (0.80 gestation), preterm delivery was induced in all of the pregnant goats (n=32) by paralumbar cesarean section under epidural anesthesia with concurrent maternal sedation and incisional infiltration with local anesthetic^(14,15)^. Sedation with xylazine (0.25 mg/kg i.m.) and sacrococcygeal epidural anesthesia into the sacrococcygeal space with a 6-cm, 20-gauge spinal needle using injection of 25 mg lidocaine hydrochloride and 0.016 mg epinephrine were performed. Then, the whole abdominal area was cleansed with 10% povidine iodine solution. A paralumbar skin incision of approximately 10 cm in length was used to reach the uterus, which was opened from its dorsal curvature with extension of the uterine incision using scissors as necessary. Before amniotomy, a 10 mL sterile syringe was used to aspirate the amniotic fluid for sampling. Then, the fetus and the placenta were delivered, and an intact placentome was dissected and sampled. The uterus was comprehensively lavaged with sterile saline solution to clean of all blood clots and membranes. Then, the uterus and the abdominal wall were closed with polyglactin 910 (Vicryl) interrupted sutures, and standard wound dressing was applied. Postpartum does were given systemic analgesic treatment with metamizole sodium and combined antibiotic treatment with 200 mg i.m. procaine penicillin and 250 mg i.m. dihydrostreptomycine sulfate.

The neonate kids were dried, weighed, and subjected to euthanasia with 50 mg/kg of intraperitoneal sodium thiopental (Pental Sodyum, IE Ulugay, Istanbul, Turkey) followed by transthoracic intracardiac blood sampling. Then, neonatal chest and skull were opened for en bloc dissection of the lung and brain. Parenchymal tissue from the lungs and white matter from the brains were sampled^(14)^. Tissue samples were fixed in 10% buffered formaldehyde and embedded into paraffin.

### Evaluation of the Samples and Immunohistochemistry

Double-antibody sandwich enzyme-linked immunosorbent assay was used to evaluate interleukin-1 (IL-1), IL-4, IL-6, and TNF-α levels in the amniotic fluid and neonatal plasma samples, using commercial kits for goat serum (Eastbiopharm, Hangzhou, China). Results were evaluated at 450 nm, and optic density values were calculated and standardized accordingly.

Immunochemical staining on fetal lung, fetal brain and placental tissues were carried out using a routine streptavidine-biotin peroxidase technique^(15)^. After primary antibody incubation, streptoavidine peroxidase incubation of the slides for 20 minutes was carried out followed by washing with phosphate-buffered saline (PBS) biotinilated Abs for 30 min. Then, PBS was used to rinse the slides, and a peroxidase substrate solution containing 3,3′-Diaminobenzidine (DAB Substrate Kit, ab94665, Abcam, UK) was used for 5 minutes of incubation. Then, rinsing with distilled water, counterstaining with hematoxylin, dehydration, and mounting of the slides were performed.

All tissues were immunostained for IL-1, IL-4, IL-6, TNF-α, caspase 3, caspase 5, caspase 7, cyclo-oxygenase-1 (COX-1), COX-2, IFN-alpha (IFN-α), and IFN-beta (IFN-β) (Abcam, UK). Additional immunostaining included surfactant proteins (SP) A, B, C, and D (Santa Cruz Biotechnology Inc., USA), and prosurfactant protein B (pro-SP-B, Abcam, UK) for the lung tissues and neuron specific enolase (NSE, Abcam, UK), glial fibrillary acidic protein (GFAP, Abcam, UK), vimentin (Abcam, UK), anti-neurofilament protein (NFP, Abcam, UK), and anti-myelin basic protein (MBP, Abcam, UK) for the brain tissues.

Degree of immunostaining was assessed by the pathologists blindly, concerning experimental groups with an arbitrary visual scale that graded the immunoreaction as 0, no staining; 1, weak staining; 2, moderate staining; and 3, diffuse staining. For semiquantitative evaluations, Database Manual Cell Sens Life Science Imaging Software System (Olympus Corporation, Tokyo, Japan) fitted with a light microscope (Olympus CX41) was utilized.

### Statistical Analysis

Data were expressed as mean and standard deviations. Kruskal-Wallis H test and Mann-Whitney U test were used for multiple and binary comparisons, respectively. Statistical significance was set at p<0.05. Kruskal-Wallis H test p-values were shown in the tables, whereas Mann-Whitney U test results for between-group comparisons were given in the text in more detail.

## Results

The mean neonatal weight was 1.453±260 g, and was similar across the groups (p=0.62). Similarly, the fetal gender was distributed equally across the groups (p=0.09). Comparisons of group 1 (vehicle control) and group 2 (fetal injury model by maternal G-CSF and high-dose IA endotoxin) have been specified in detail in our previous publication^(14)^ with validation of the current model of intrauterine inflammation. Briefly, maternal G-CSF and IA endotoxin led to increased IL-1, TNF-α, IFN-α, and IFN-β, COX-1, COX-2, caspase 3, 5, and 7, and reflex increase in IL-4 and IL-6 in all of the evaluated tissues along with decreased pulmonary SP-A, SP-B, SP-C, SP-D, pro-SP-B, and decreased brain vimentin, neuron specific enolase (NSE), neurofilament protein (NFP), GFAP, and myelin basic protein (MBP) expressions.

### Amniotic Fluid Inflammatory Markers

**Figure 1:**
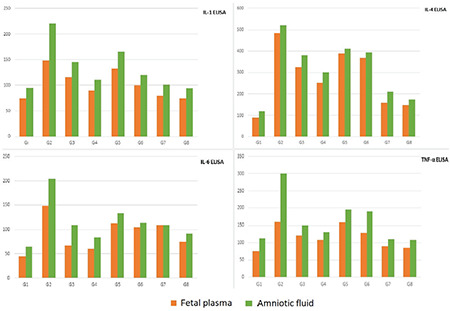
Fetal blood plasma and amniotic fluid interleukin-1, interleukin-4, interleukin-6, and tumor necrosis factor-alpha measurements obtained at preterm delivery in experimental study groups

Data on amniotic fluid sampled at preterm cesarean delivery are given in Table 1 and summarized in Figure 1. Both low-and high-dose oral PTX regimens significantly lowered IA IL and TNF-α levels compared to the model group (p=0.03 for both comparisons), However, reflex increase in immunomodulatory interleukins was more efficiently suppressed with high-dose oral PTX (group 4) compared to the low-dose (group 3), considering IL-4 and IL-6 expressions (p=0.04 for all comparisons). High-dose oral administration was able to partially reverse IA inflammation with comparable IL-1 (p=0.15) and IL-6 (p=0.07) values across group 1 (vehicle controls) and group 4.

Both low-and high-dose IA PTX were also effective in reducing IA inflammation with decreased IA ILs and TNF-α compared to group 2 (p=0.03 for all comparisons except p=0.07 for low-dose IA PTX). The anti-inflammatory effect seemed similar between two IA doses (p>0.05 for all comparisons across group 5 and group 6). Despite this, IA PTX administrations were not able to completely reverse the IA inflammation, since comparisons of all parameters between IA PTX treatment groups and vehicle controls showed statistically significant differences (p=0.03 for all). Standalone IA PTX treatment (group 7 and group 8) was generally associated with similar IA inflammatory marker values (p>0.05) except increased IL-6 (p=0.02) with low-dose PTX (group 7) compared to group 1 controls.

The current data underpinned the anti-inflammatory effects of both prophylactic oral and therapeutic IA PTX, although high-dose oral protocol showed a more robust activity to alleviate endotoxin-induced IA inflammation (Figure 1).

### Neonatal Plasma Inflammatory Markers

Comparisons of the studied inflammatory parameters in the neonatal blood plasma obtained following preterm delivery are summarized inTable 1 and Figure 1. Low-dose oral PTX was associated with decreased IL-1, -4, and -6 levels in the neonatal plasma (p=0.03 for all), although TNF-α was unchanged (p=0.07), when compared with vehicle controls (Table 1). High-dose oral PTX seemed more effective in reducing circulating inflammatory markers, with all of the studied ILs and TNF-α significantly less than that of group 2 (p=0.03 for all comparisons). However, only IL-1 measurements after high-dose oral PTX were similar (p=0.07) to normal controls, indicating a partial reversal of inflammation following the high-dose oral regimen.

For IA treatment with PTX, high-dose (group 6) decreased all of the studied neonatal plasma ILs (p=0.03 for all comparisons), but not TNF-α (p=0.07) compared to endotoxin controls (group 2). However, only mean IL-1 level was similar (p=0.07) to that of group 1 controls, indicating a partial reversal of inflammation with the high-dose administration. Low-dose IA PTX (group 5) was less effective with only IL-4 levels significantly decreased (p=0.04) in the neonatal plasma compared to endotoxin controls. Despite these positive effects, standalone IA PTX was associated with increased IL-6 plasma measurements following both low-dose and high-dose IA PTX (p=0.02 and p=0.03, respectively) compared to vehicle controls.

As a result, both high-dose oral and high-dose IA PTX effectively reduced the studied inflammatory markers in the neonatal plasma, but did not completely reverse the fetal inflammation back to control levels. A possible adverse effect of IA PTX may be stimulation of an immune reaction through IL-6 in the fetus (Figure 1).

### Placental Immunohistochemical Findings

Table 2 summarizes comparisons of placental immunostaining intensities between the study groups. Group 2 (model) was associated with profound placental inflammation and apoptosis, as previously reported^(14)^. Low-dose oral PTX (group 3) partially reversed the findings, as shown by decreased IL-1 (p=0.02), IL-6 (p=0.04), caspase 3 (p=0.02), and COX2 (p=0.04) expressions compared to endotoxin exposed controls (group 2). High-dose oral PTX (group 4) alleviated placental inflammation and apoptosis to a great extent with return to baseline expressions (p>0.05) except COX2 (p=0.01 for group 1 and group 4 comparison).

**Figure 2:**
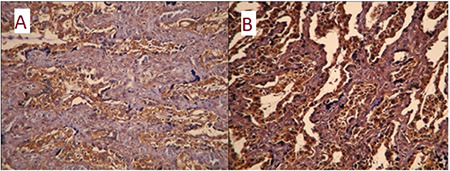
Placental caspase-7 immunoreaction in (A) group 6 and (B) group 7 that received intra-amniotic pentoxifylline with or without intra-amniotic endotoxin, respectively. Administration of pentoxifylline directly into the amniotic fluid could trigger apoptosis in the placenta

Although low-dose IA PTX (group 5) led to decreased expressions of some inflammatory parameters in the placenta (IL-1, IL-6, IFN-α, and COX2 with p=0.01, p=0.04, p=0.04, and p=0.04, respectively) compared to positive controls, high-dose IA PTX (group 6) seemed ineffective (p>0.05 for all comparisons between group 2 and group 6). Standalone low- and high-dose IA PTX was associated with increased caspase 3 (p=0.04 and p=0.05, respectively) and caspase 7 (p=0.03 and p=0.05, respectively) expressions in the placenta, indicating a possible apoptotic process in the placenta following PTX injection into the amniotic fluid (Figure 2).

These results revealed that prophylactic oral PTX prevented placental inflammation and apoptosis, and high-dose oral regimen was probably more effective with return to baseline levels except COX2. On the other hand, IA PTX was less effective and may be associated with placental apoptosis.

### Fetal Brain Immunohistochemical Findings

Table 3 summarizes the immunostaining intensities of the fetal brain white matter. As previously shown^(14)^, inflammatory and apoptotic markers were increased (p<0.05), leading to fetal brain injury apparent with decreased NSE, NFP, GFP, and MBP staining in endotoxin-exposed positive controls (group 2). Low-dose oral PTX (group 3) was associated with decreased IL-1 (p=0.02), IFN-β (p=0.03), caspase 3 (p=0.03), COX1 (p=0.02), and increased NSE (p=0.03) staining compared to positive controls (group 2). On the other hand, high-dose oral PTX (group 4) had a more profound effect, leading to significantly improved expressions (p<0.05) of all of the parameters except COX-2 (p=0.06), GFAP (p=0.06), and MBP (p=0.06). There were no significant differences (p>0.05 for all comparisons) between group 4 and vehicle controls (group 1), indicating reversal of the brain injury with high-dose oral PTX.

Low-dose IA PTX (group 5) led to decreased IL-1 (p=0.018), IFN-α (p=0.04), IFN-β (p=0.04), and TNF-α (p=0.02) immunostaining in the fetal brain compared to endotoxin controls (group 2). Following high-dose IA PTX (group 6), only IL-1 (p=0.02), and IFN-β (p=0.04) staining were reduced. Low- and high-dose IA PTX (group 5 and group 6) were similar (p>0.05 for all comparisons) considering all parameters. Low-dose PTX administration into the amniotic fluid without fetal injury (group 7) was associated with increased caspase 3 (p=0.02) and caspase 5 (p=0.04), and decreased NSE (p=0.02), whereas high-dose (group 8) led to decreased vimentin and NSE (p=0.02 for both) compared to vehicle controls (group 1).

Therefore, high-dose oral PTX seemed more effective than low-dose oral PTX against fetal brain injury. The results also indicated some anti-inflammatory effect of IA PTX on the preterm fetal brain, although PTX might lead to increased white matter apoptosis and neuronal injury, when injected into the amniotic fluid.

### Fetal Pulmonary Immunohistochemical Findings

Table 4 shows the immunostaining intensities of the fetal pulmonary parenchyma. As expected, inflammatory and apoptotic parameters were increased, whereas surfactants were decreased in the model group (group 2). Low-dose oral PTX (group 3) alleviated IL-1 (p=0.02), IL-4 (p=0.04), IFN-α (p=0.02), IFN-β (p=0.03), caspase 3 (p=0.03), caspase 5 (p=0.03), and COX1 (p=0.02) expressions compared to positive controls (group 2). High-dose oral PTX (group 4) was more effective, as shown by amelioration of all lung parameters to baseline (group 1 control) levels (p>0.05 for all comparisons) and significantly decreased inflammation and apoptosis (p<0.05 for all) except COX2 staining (p=0.07) with increased surfactant protein levels (p=0.02 for all).

IA PTX was generally associated with improved fetal pulmonary inflammation; however, lacked any positive effects on apoptosis and surfactant synthesis. Both low- and high-dose IA PTX (group 5 and group 6) decreased pulmonary IL-1 (p=0.02 for both doses), reflex increase in IL-4 (p=0.02 for both doses), and IFN-α staining (p=0.01 and p=0.02, respectively) compared to endotoxin-exposed controls (group 2). High-dose was also associated with decreased pulmonary COX1 expressions (p=0.02). No significant differences were present for other parameters, when group 5 or 6 were compared with group 2. Standalone (i.e. without endotoxin) IA PTX at low (group 7) or high (group 8) doses did not generally alter immunostaining of the studied parameters (p>0.05 for all comparisons with group 1 controls), demonstrating that PTX did not have any unwanted effects on fetal lungs after IA administration.

Overall, high-dose oral PTX was the most effective treatment modality against inflammation-driven preterm fetal pulmonary injury with return to baseline expressions except COX2.

## Discussion

In the current study, we tested the therapeutic efficacy of oral and IA PTX administrations against inflammation-mediated placental and fetal injury in the preterm goat model. Oral PTX was given prophylactically (i.e. before IA endotoxin injection), whereas IA PTX was administered concomitantly with endotoxin. Two different doses labeled as low- and high-dose treatments were evaluated for both the oral and IA ways of administration. Overall data indicate high-dose oral (60 mg/kg maternal weight daily) use of PTX for 15 days prior to IA endotoxin insult as the most effective protective therapeutic option against inflammation-driven fetal injury. Although PTX when given into the amniotic fluid had some potential to decrease the IA inflammation, some adverse effects such as increased apoptosis in the placenta and fetal brain secondary to stimulation of an immune or toxic reaction is a concern that may restrict use of IA PTX for preterm fetal neuroprotection.

There are some potential explanations why oral PTX was more effective in our design. Since the oral protocol was prophylactic in nature with repeated daily doses, the neuroprotective effects of PTX might have become more apparent before the inflammatory insult. This may imply that PTX is generally active as a preventive therapy, when given relatively early in the inflammation-driven preterm birth model with limited action following delayed administration. Repeated doses of PTX instead of a single-dose may be required for relevant action, as well. However, maternal and placental pharmacokinetics of PTX may also play a role. Oral PTX administered maternally is metabolized into at least 7 nonconjugated metabolites by the liver, and the main active metabolite called lisofylline has been shown to have anti-inflammatory and antifibrotic activity^(20,21)^. Moreover, the anti-inflammatory actions of both PTX and lisofylline were attributed to 8-oxo derivatives rather than the parent forms^(22)^. Since administration of PTX directly into the amniotic fluid will not enable hepatic metabolization of PTX into active forms, IA drug delivery may have inadequate anti-inflammatory and fetal neuroprotective actions. Furthermore, it can be speculated that the maternal liver and possibly the placenta “detoxify” the parent compound into more active and less fetotoxic compounds. Evidence in favor of such an effect can be adverse fetal reactions we encountered in our design, including increased apoptosis in fetal brain following IA PTX administrations in pregnancies without intrauterine inflammation. In summary, oral PTX administered to risk-groups for preterm delivery should be the preferred way of administration for fetal neuroprotection.

Experimental data on use of PTX for inflammation-induced adverse effects in the placenta are scarce. In a tissue culture model that used second trimester human placentas treated with endotoxin and PTX, placental expression and production of inflammatory markers such as IL-1, TNF-α, and IFN were shown to decrease with administration of PTX^(23)^. These *ex vivo* results are in agreement with *in vivo* data from our experiments, affirming the anti-inflammatory actions of PTX on the placenta.

Some previous animal studies have also evaluated the placental and fetal effects of maternal PTX in the endotoxin-induced intrauterine inflammation models. PTX was reported to mitigate endotoxin-induced up-regulation of placental heme oxygenase-1 in pregnant mice^(24)^. PTX was also shown to decrease embryo resorption, fetal mortality, and fetal growth restriction following lipopolysaccharide injections to pregnant mice^(25,26)^. However, these positive effects were only partially replicated in a rabbit model. In pregnant rabbits given intrauterine endotoxin, animals that received PTX 20 mg/kg/day in 3 divided doses had similar preterm delivery rates, despite prolonged time until fetal death compared to controls^(27)^. On the other hand, in experimentally induced equine placentitis using intracervical inoculation of *Streptococcus equi*, 17 mg/kg daily maternal dose of PTX given orally from the onset of clinical signs to delivery was associated with improved viability of foals and negative fetal bacterial cultures^(28)^. Depending on these results in association with our current data, oral maternal PTX treatment given for a longer period, particularly before placental and fetal injury commences seems more effective to alleviate subsequent intrauterine inflammation.

In the English literature, we were able to identify only one experimental study that specifically evaluated the fetal neuroprotective effects of antenatally administered PTX. This study by Dilek et al.^(29)^ used intraperitoneal endotoxin to induce fetal injury in pregnant rats, and 3 doses (60 mg/kg) of maternally injected PTX before term delivery was associated with decreased apoptosis and MBP immunostaining in periventricular white matter of the pups. The results imply that PTX is a potential neuroprotective agent against fetal term brain injury^(29)^. Our data from a phylogenetically diverse animal model in the early preterm period with numerous inflammatory and neuronal injury markers elaborate these previous findings.

In summary, previous experimental data show that prophylactic maternal PTX treatment may decrease placental and fetal brain injury following an intrauterine inflammatory insult, but probably do not prevent preterm delivery. Although we did not specifically address the timing of preterm delivery in our experiments, our results generally elaborate these findings and support use of maternal PTX as a fetal neuroprotective agent, particularly for pregnancies at risk for preterm delivery. Moreover, we showed the efficacy of oral daily doses at 60 mg/kg, which is clinically more feasible than parenteral injections.

We could identify only one clinical study on the use of antenatal PTX in human pregnancies. The randomized prospective trial by Lauterbach et al.^(30) ^included 96 women between 23-34 weeks of gestation with imminent preterm delivery, randomized to 800 mg/day PTX (n=43) and controls (n=53) for 3 weeks or until delivery, with all the women receiving standard beta-mimetic tocolysis, corticosteroids for fetal lung maturation, and magnesium sulfate for neuroprotection. The cerebroplacental ratio at week 3 of treatment was found to be significantly higher in the treatment arm. Moreover, composite neonatal outcome (intraventricular bleeding, periventricular leukomalacia, and neonatal mortality) was lower in the PTX arm, although time to delivery was similar. Our results are principally in line with the findings from this pilot clinical study^(30)^. We did not specifically evaluate fetal Doppler studies or neonatal mortality. However, our results similarly imply that PTX given for at least 2 weeks has fetal neuroprotective effects. Lauterbach et al.^(30)^ administered half of the daily 800 mg dose as an intravenous infusion, and the rest 400 mg as oral tablet. Considering the dose scheme in our study that corresponds to higher oral doses than that of Lauterbach et al.^(30)^, the necessity and feasibility of additional daily intravenous administrations are questionable. Therefore, future clinical studies evaluating fetal neuroprotective effects of prophylactic antenatal PTX therapy can focus on relatively low (800-1600 mg) daily oral doses of PTX without any parenteral administrations, which necessities admission to the hospital.

### Study Limitations

There were certain limitations of the current experimental study. Although various tissue parameters for inflammation, apoptosis, and tissue injury were studied by immunohistochemistry, additional methods such as genetic expressions and Western blot were not evaluated. Since there is positive experimental and clinical evidence in favor of safety of oral PTX use during the third-trimester of pregnancy, our design did not include standalone oral PTX experimental groups. There was also no oral placebo group. However, addition of more experimental groups into the present design would be unethical. C-reactive protein and white blood cell count and differential in fetal blood samples were not measured, as interleukins and TNF-α were considered more specific for fetal inflammatory response. The current design was also unable to reveal data on temporal changes of the studied parameters. In our validated model, preterm birth was induced 5 days after IA endotoxin, a time period supposed to be most suitable for evaluation of fetal inflammatory response. However, we do not have data on other time points, including postpartum alterations, since newborn kids were euthanized after delivery to illustrate specifically *in utero* tissue responses. The present experimental model cannot also distinguish whether the experimental IA inflammation created in the intervention groups in the study essentially caused spontaneous preterm birth, since preterm delivery was induced iatrogenically to retrieve fetal and placental samples. Finally, CP is multifactorial condition that can occur during the intrapartum period, during, or after delivery. The current data do not explicitly reveal information on preterm birth related to CP and/or related complications, and are restricted to experimental fetal injury in the preterm period with lack of analysis of mid- and long-term results of CP.

## Conclusion

Maternal PTX given orally before inflammation-mediated preterm delivery in a prophylactic fashion alleviates fetal inflammatory response and may exhibit fetal neuroprotective effects in the caprine model. Considering the favorable fetal safety profile of PTX during the third trimester of pregnancy, clinical studies evaluating its antenatal use in imminent preterm delivery against subsequent development of possible brain injury are warranted.

## Figures and Tables

**Table 1:**
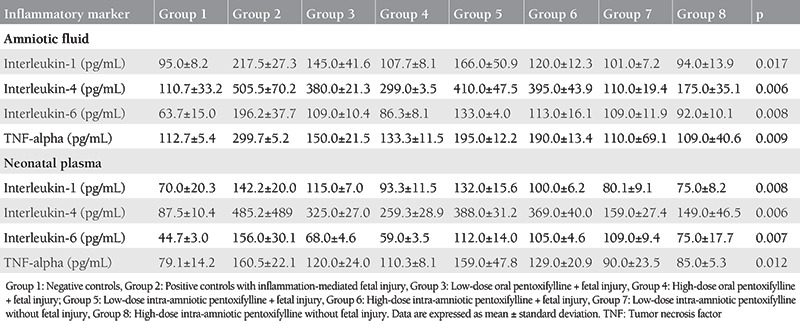
Comparisons of amniotic fluid and neonatal plasma inflammatory markers between experimental groups (n=4 in each group) in preterm caprine pregnancies

**Table 2:**
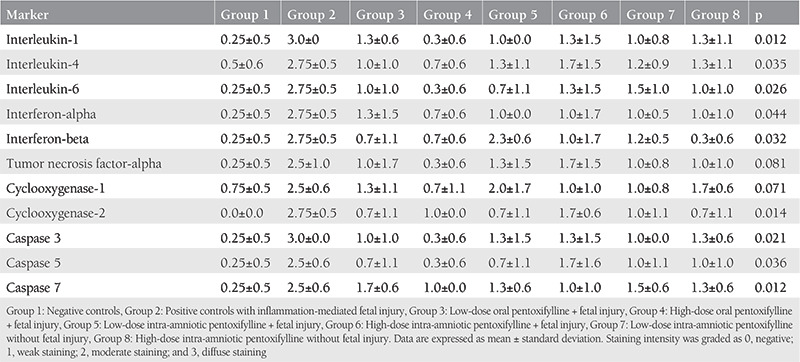
Comparisons of placental immunostaining intensities between experimental groups (n=4 in each group) of preterm caprine pregnancies

**Table 3:**
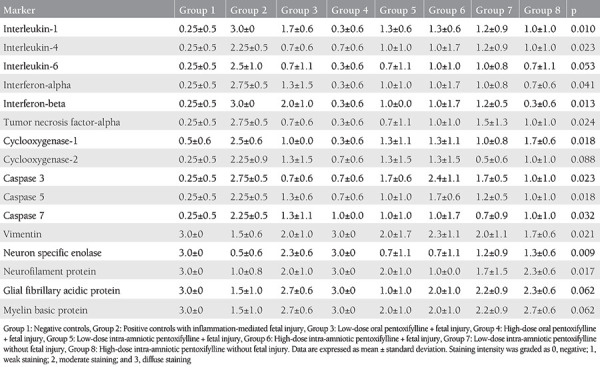
Comparisons of fetal brain white matter immunostaining intensities between experimental groups (n=4 in each group) of preterm kids

**Table 4:**
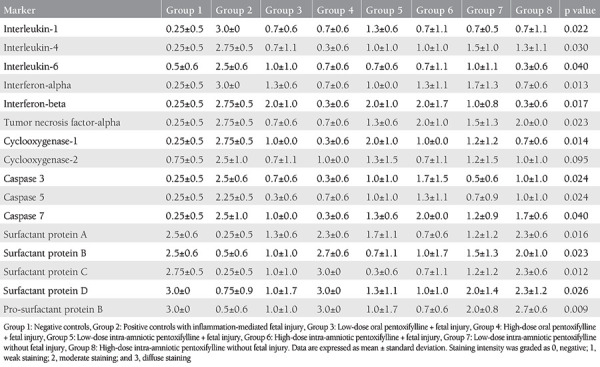
Comparisons of fetal pulmonary parenchymal immunostaining intensities between experimental groups (n=4 in each group) of preterm kids
